# Transmembrane Protein Docking with JabberDock

**DOI:** 10.1021/acs.jcim.0c01315

**Published:** 2021-02-26

**Authors:** Lucas S. P. Rudden, Matteo T. Degiacomi

**Affiliations:** † Department of Physics, Durham University, South Road, DH1 3LE Durham, United Kingdom

## Abstract

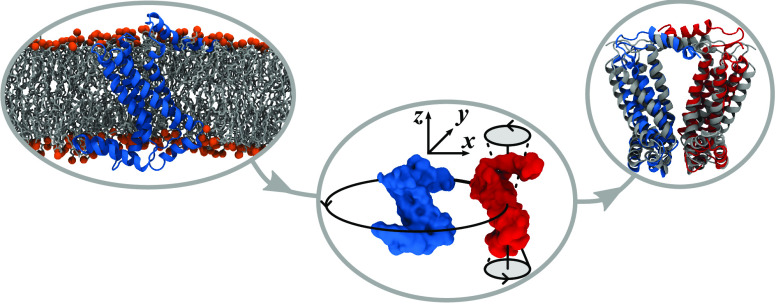

Transmembrane proteins act as an intermediary for a broad range of biological process. Making up 20% to 30% of the proteome, their ubiquitous nature has resulted in them comprising 50% of all targets in drug design. Despite their importance, they make up only 4% of all structures in the PDB database, primarily owing to difficulties associated with isolating and characterizing them. Membrane protein docking algorithms could help to fill this knowledge gap, yet only few exist. Moreover, these existing methods achieve success rates lower than the current best soluble proteins docking software. We present and test a pipeline using our software, JabberDock, to dock membrane proteins. JabberDock docks shapes representative of membrane protein structure and dynamics in their biphasic environment. We verify JabberDock’s ability to yield accurate predictions by applying it to a benchmark of 20 transmembrane dimers, returning a success rate of 75.0%. This makes our software very competitive among available membrane protein–protein docking tools.

## Introduction

Transmembrane proteins play an essential role as a mediator for many functions critical to an organism’s survival. Situated within a lipid membrane that compartmentalizes two distinct biological regimes, their tasks include sensing, signaling, motility, endocytosis, and anchoring. Their malfunction is responsible for a multitude of diseases,^1^ and consequently, they are a frequent target in drug design. The formation of complexes, wherein two or more transmembrane proteins will oligomerize into either helix bundles or β-barrels, is of vital significance to both the function and malfunction of these processes. Yet, of the ∼170,000 structures available on the PDB database, only ∼7000 (4%) are transmembrane proteins^2^ despite them making up 20%–30% of the proteome and 50% of all known drug targets.^3^ This relatively small number of available structures is primarily due to the greater technical difficulties associated with characterizing them compared to soluble proteins. A computational tool capable of accurately predicting complexes would therefore help address some of this knowledge gap, provide understanding to underlying biological mechanisms, and inform drug design.

A plethora of increasingly sophisticated protein–protein docking approaches have been developed to address the problem of protein assembly prediction.^4^ These efforts are nucleated around the community-led CAPRI competition, which is used to identify the most reliable algorithms, promising methodologies and current hurdles.^5^ However, the vast majority of these methods center around the docking of two or more soluble proteins. While docking transmembrane proteins is facilitated by limitations on the search space imposed by the lipid bilayer, membrane docking algorithms must consider the impact of the lipid bilayer on a protein’s recognition of a partner in tandem with the solvent. In this context, there are only a small number of tools currently available. MPDock,^6^ utilizing existing Rosetta sampling and scoring methods in an integrative modeling context, found a successful high ranking pose in three out of five applied bound complexes. Hurwitz et al.’s program Memdock^7^ uses a traditional rigid docking, refinement, reranking method, with energetic terms representing the membrane’s hydrophobic environment included in the final stage. Comparing the performance of Memdock and GRAMM-X^8^ on 11 unbound complexes, the authors showed that the first yielded a success rate of 36.4% and the latter of 9.1%. Viswanath et al. used the DOCK/PIPER^9^ docking algorithm with an additional reranking step that considered the membrane transfer energy, achieving a success rate of 36.6% for 26 unbound complexes. Testing other software on the same data set, the authors reported success rates of 30%, 46.6%, and 56.6% for ZDOCK+ZRANK,^10,11^ CLUSPRO,^12^ and GRAMM-X,^8^ respectively. All of these approaches were only tested against cases featuring α-helical transmembrane proteins. Koukos et al., using HADDOCK^13^ without any specific membrane protein optimization, achieved a blind docking success rate of 19.2% on their dimeric unbound data set of 26 complexes that included β-barrel, monotopic, and α-helix proteins. Of these 26 test cases, 11 featured a pair of integral proteins as binding partners, and only three of these were unbound-ligand-to-unbound-receptor docking. The latter achieved a success rate of 36.4%. HADDOCK has also very recently been combined as a refinement tool with the LightDock^14^ docking algorithm and tested against 18 transmembrane-soluble protein complexes,^15^ achieving a success rate of 61.1%. At the time of publication, MPDock was presented as a proof of concept, not yet designed for widespread use. The DOCK/PIPER membrane energy reranking tool is available for download, but it must be applied to models obtained independently. Memdock is usable as a web server, although requires input structures to have their solvent-exposed regions manually removed.

We recently released our protein–protein docking software, JabberDock,^16^ after testing it against a standard benchmark of 226 soluble complexes developed by the CAPRI community.^17^ It obtained a greater than 54% success rate, with the notable achievement that the flexibility of the individual structures made little difference to its overall success. JabberDock’s defining feature is its usage of a novel protein volumetric representation called spatial and temporal influence density (STID) maps, which are built from short molecular dynamics (MD) simulations. STID maps are generated via a physical model describing the protein’s shape and electrostatic and residue-level dynamics. Through a comprehensive benchmark, we identified an ideal cutoff value (isovalue) to transform STID volumetric maps into three-dimensional surfaces. JabberDock docks proteins represented by these shapes, attempting to maximize their surface complementarity. A key characteristic of STID maps is that the ideal isovalue to transform them into shapes emerges naturally from the MD simulation, specifically from the relationship between the solvent accessible surface area and the average STID value. Thus, crucially, it is environmentally independent. This property makes STID maps an attractive representation for membrane proteins, exposed to a biphasic environment. The STID map representative of a transmembrane protein can be obtained by independently simulating the preoriented partners immersed in an explicit lipid bilayer. Docking then requires maximizing the complementarity of two membrane protein surfaces, with the ligand’s translational motion perpendicular to the membrane and rotations into the plane of the bilayer constrained. Preliminary work in this endeavor yielded encouraging results: we predicted the transmembrane dimeric complex formed by *bo*_3_ oxidase with our top-scoring pose corroborating available mass photometry data.^18^ Herein, we present and test our methodology to dock integral membrane protein dimers, now available in JabberDock as an automated pipeline.

## Results

**Figure 1:**
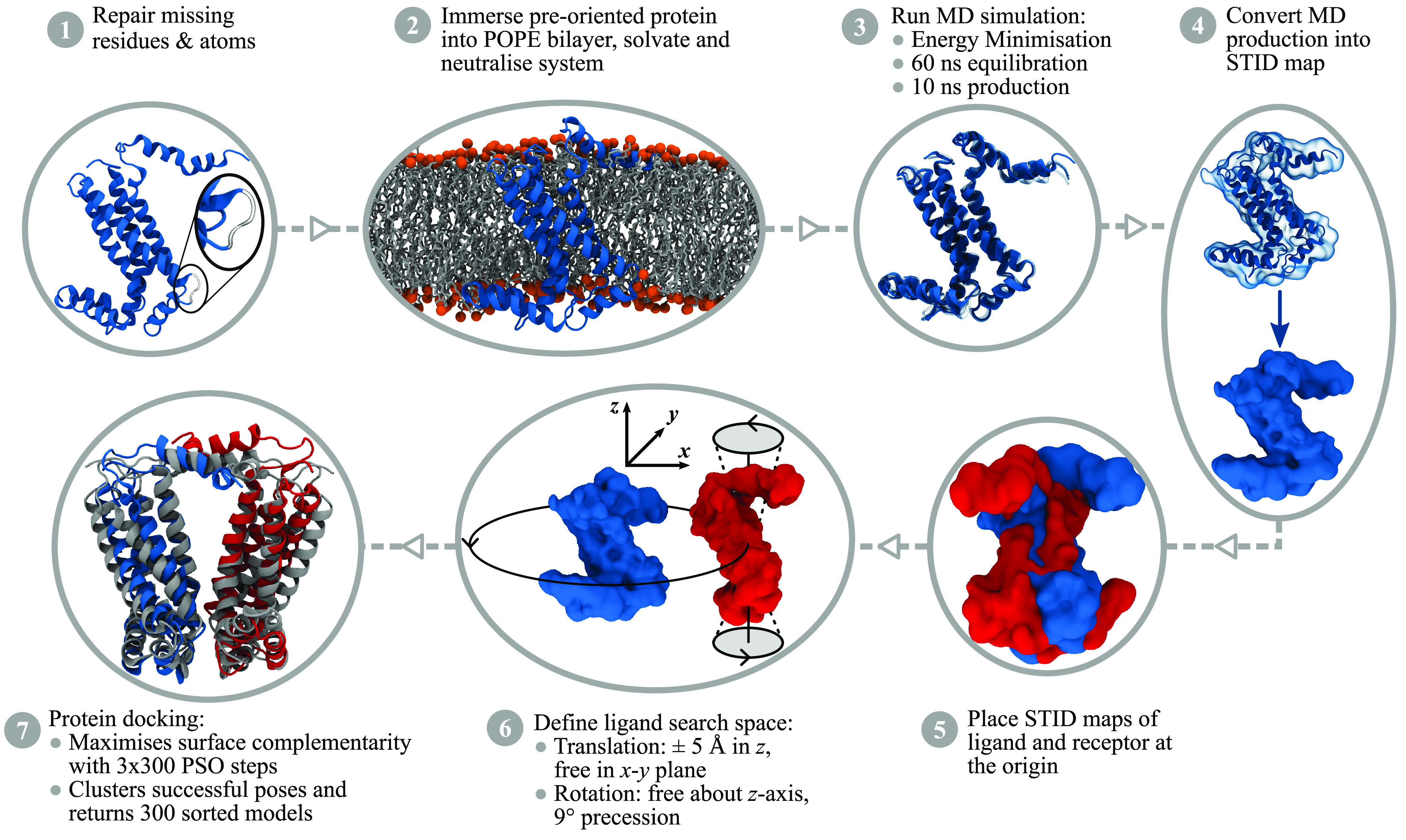
JabberDock transmembrane protein docking pipeline. Full details of each step, including a convergence benchmark for Step 4, are available in Methods. This example’s target complex is the homodimer 1Q90 (BF), using 2ZT9 (A) as the ligand (blue) and receptor (red). Step 7 features a representation of the fifth best model; an intermediate success overlaid on the bound structure (gray).

JabberDock docks transmembrane proteins via a multistage process summarized by the flow diagram in Figure 1 and fully detailed in Methods. In short, JabberDock requires input protein structures to be aligned with the center of mass for the transmembrane region of the proteins at *z* = 0, where the *z*-axis is perpendicular to the bilayer plane. In our tests, we obtained these preorientated structures via the OPM server.^19^ Structures are repaired where necessary using the Modeller package,^20^ before being immersed in a POPE bilayer via the PACKMOL-memgen tool.^21^ GROMACS^22^ is then used to generate the simulation data using the Amber14SB^23^ and SLipid^24^ force fields, which enables the generation of STID maps. The maps of both binding partners are then converted into isosurfaces using a predetermined cutoff and docked such that their surface complementarity is maximized^16^ (see SI and Figure S2). This surface-based scoring function is effective because it bypasses the need to explicitly handle packing of interfacial atoms, yielding smoother and gentler gradients compared to typical atomistic representations (see SI and Figure S3). Here, the search space is navigated using the particle swarm optimisation algorithm implemented in the POWer optimization engine.^25^ On average, our full docking pipeline requires 3 days for simulation and 12 h for docking on our hardware (see details in SI).

There does not yet exist a standard transmembrane protein docking benchmark equivalent to the soluble proteins one made available by the CAPRI community.^17^ To test JabberDock, we selected all the unbound cases involving pairs of transmembrane proteins within Memdock,^7^ HADDOCK,^13^ and DOCK/PIPER^9^ benchmarks. To avoid testing against similar examples, and thus biasing our statistics, we only selected one representative within test cases featuring greater than 80% sequence homology. This resulted in a diverse benchmark set featuring 20 α-helical complexes. We summarize our results for each test case in Table 1. Full details, including the three metrics used to define success by the CAPRI community (RMSD of the best pose, with its corresponding ratio of correct residue contacts (*f*_nat._) and interfacial RMSD), are given in Table S1, spreadsheet.

**Table 1:** Results of Membrane Docking Benchmark^a^

Target	Receptor	Ligand	Rank of first successful model	Quality of best pose in top 10
1BL8 (AB)	1K4D (C)	1K4D (C)	2	∗∗
1EHK (AB)	3S33 (A)	3S33 (B)	1	∗
1H2S (CD)	1GU8 (A)	2F95 (B)	1	∗∗
2WIE (AB)	3V3C (A)	3V3C (A)	1	∗∗
1E12 (AC)	3A7K (A)	3A7K (A)	2	∗
1M56 (AC)	3OMI (A)	1QLE (C)	X	–
1Q90 (BF)	2ZT9 (A)	2ZT9 (A)	5	∗∗
1ZOY (CD)	1YQ3 (C)	1YQ3 (D)	159	–
2QJY (AD)	1ZRT (C)	1ZRT (C)	3	∗∗
3CHX (BJ)	1YEW (B)	1YEW (B)	8	∗
3KLY (AB)	3KCU (A)	3KCU (A)	5	∗∗
3OE0 (AB)	3ODU (A)	3ODU (A)	X	–
3RVY (AB)	3RW0 (A)	3RW0 (A)	X	–
4DKL (AB)	4EA3 (A)	4EA3 (A)	1	∗∗
2NRF (AB)	2IC8 (A)	2IC8 (A)	1	*
2VT4 (AB)	2Y00 (A)	2Y00 (B)	8	∗∗
3KCU (AB)	3Q7K (A)	3Q7K (A)	1	∗
1M0L (AC)	1C8S (A)	1C8S (A)	7	∗
2K9J (BA)	2RMZ (A)	2K1A (A)	1	∗
2KS1 (BA)	2N2A (A)	2M0B (A)	135	–

a The target complex is provided with two composite chains (name indicated in parentheses), which the receptor and ligand correspond to respectively. The rank of the first successful model, either of acceptable (∗) or intermediate (∗∗) quality as determined by the CAPRI criteria (see Methods), is given along with the quality of the best pose found in the top 10 predictions. X indicates that no successful pose was found within the 300 models produced. See Table S1, spreadsheet, for details.

**Figure 2:**
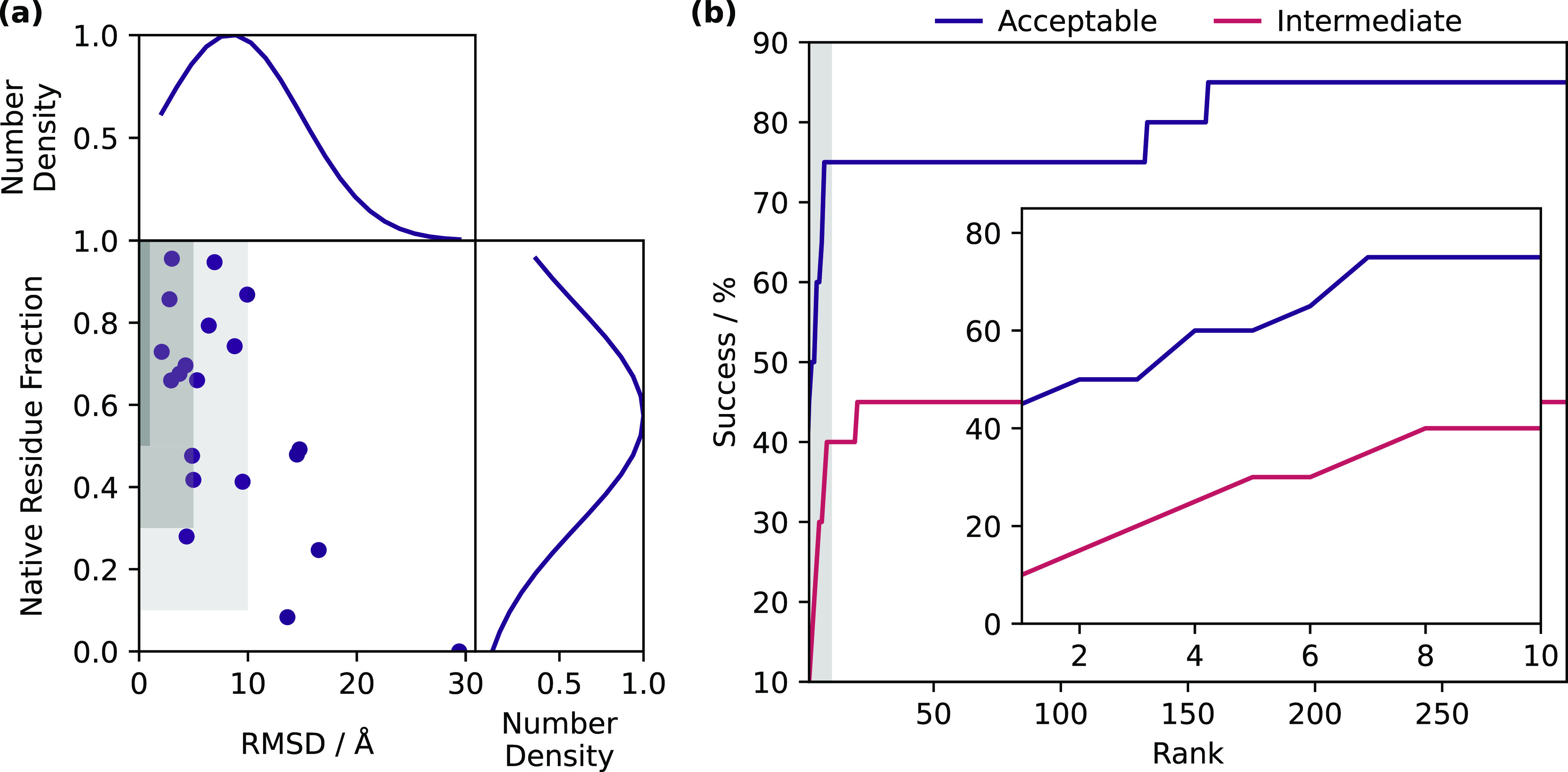
(a) Quality of best models within the top 10 results for every docking case. For each case, the lowest α-carbon RMSD between the prediction and crystallized homologue is presented against the associated native residue fraction (*f*_nat._). The dark- to light-shaded regions represent the criteria for high to acceptable quality results. Thus, a point landing in one of these regions indicates a success. (b) Percentage of cases yielding an acceptable (purple) and intermediate (pink) success as a function of the number of ranked structures considered as candidate models. The region corresponding to the top 10 models is shaded and magnified in the inset.

JabberDock was successful (i.e., yielding at least one acceptable model or better among its top 10 candidates) in 75% of the cases in our benchmark set, producing an intermediate quality success in 40% of cases (see Figure 2, Figure S5 and Table S1). This remarkable performance is explained by JabberDock’s ability to identify the binding interface correctly, primarily due to its sensitivity to the dynamics of individual amino acids. Indeed, as shown in Figure 2a, in nearly every test case, at least one prediction in the top 10 results features a correctly identified binding interface. We also notice that results obtained here are superior to those we reported for JabberDock against soluble proteins (54%). Given that our STID map-based scoring function performs comparatively in a water and membrane environment (see Figure S2), this substantial increase can be explained by the added benefit of *a priori* knowledge about the orientation of the proteins with respect to the bilayer, coupled with the strict constraints imposed by the lipid membrane.

Expanding the pool of candidate structures to the whole 300 models returned by JabberDock does little to improve its overall success rate (with a successful model produced in 85.0% of cases, see Figure 2b), in contrast to other protein docking software and the soluble benchmark. This is because JabberDock returned a top 10 successful model for the majority of cases it dealt with (15 out of 20). The few unsuccessful cases, also challenging for other docking algorithms, possess similar structural features to those complexes in the soluble benchmark that JabberDock found problematic. The NavAb voltage-gated sodium channel (PDB: 3RVY) features an interlocked arrangement where, following the unbound MD simulation, the binding site closed up, preventing the ligand STID surface from navigating into the binding pocket. The wild type cytochrome c oxidase (PDB: 1M56) lacks characteristic surface features (i.e., it is relatively smooth), making it difficult for JabberDock to differentiate between nonbinding and binding regions (see Figure S4). All remaining cases that were not successful featured either, individually or as a combination, surfaces devoid of feature-rich regions (see SI and Figure S4), or relatively small binding interfaces, particularly demanding to identify given the goal of the optimizer to maximize surface complementarity.

Some proteins can form multiple complexes by interacting with different binding partners. In our previous work,^16^ we observed that knowledge of a protein’s bound state with a specific partner may facilitate its docking with a different one; i.e., the bound state of the native complex from which the ligand or receptor is sourced can be used as a surrogate for the target complex. Here, we tested this approach with the 1M56 test case, comprised of two binding partners that have had their structures solved as part of an alternative complex. As reported in Table S1, when docking STID maps generated from MD simulations of monomeric binding partners (i.e., extracted from their existing complexes and simulated in the unbound state), none of the 300 candidate models were successful. In contrast, docking STID maps generated from surrogate bound-state conformations (i.e., from simulations of alternative complexes) yielded eight successful poses, the best one at rank 51. This improvement, although not featuring a top 10 successful result, indicates that membrane protein docking may benefit from STID maps representing bound dynamics extracted from other known complexes these membrane proteins are involved with.

## Discussion and Conclusion

We have presented a pipeline enabling our blind soluble protein–protein docking software, JabberDock, to successfully tackle cases involving integral membrane protein dimers. This success is due to the molecular representation we adopt to dock proteins (STID maps), casting electrostatics, dynamics, and the protein’s shape into a single volumetric representation. The preliminary stages in the building of a STID map require an MD simulation; thus, the different characteristics expressed by the protein in both the soluble and lipid environments are encapsulated in the isosurface’s topography. Consequently, other than an extended MD simulation, one only needs to restrict the search space of the ligand in the docking protocol to regions occupied by the lipid membrane. The problem is, therefore, more manageable overall than a soluble protein docking one.

As no standard transmembrane protein docking benchmark exists, we applied JabberDock to an unbound benchmark of 20 transmembrane α-helix proteins taken from three other benchmarks,^7,9,13^ which returned a success rate of 75%. These results correspond to correctly identifying seven versus DOCK/PIPER’s two out of eight cases,^9^ eight versus Memdock’s four out of eleven cases,^7^ and one versus HADDOCK’s one out of three cases^13^ (note that two cases were tested by more than one of these methods, hence, 22 individual comparisons from 20 cases). Applying the same difficulty classification method employed by CAPRI to soluble protein docking (see Methods), we see that acceptable models within the top 10 candidates were obtained even for some of the most flexible cases. Unsuccessful cases were primarily those where the STID maps featured flat interfaces, a similar issue encountered with the soluble benchmark set. In this context, we have observed that docking binding partners using STID maps generated from alternative complexes could improve the docking quality of an otherwise unsuccessful docking case. The observed increase in docking accuracy was less significant than what we previously observed for globular proteins, where the improvement yielded several successful complexes in the top 10 predictions. This difference is potentially because the change in dynamics from switching a binding interface from lipids to a protein is smaller than the equivalent with a water solvent. It nevertheless demonstrates that there is scope for increasing JabberDock success rate by refining our STID map representation. Given the success of the results presented here and that previously demonstrated with globular proteins, we expect JabberDock to also perform well with transmembrane-solvent proteins, regardless of whether the ligand is extracellular, periplasmic, or cytoplasmic.

## Availability

JabberDock is available for download under GPL license at https://github.com/degiacom/JabberDock, along with input and target structures used in our benchmark. Authors will release the atomic coordinates of all produced models upon article publication using Durham Research Online Data sets Archive (DRO–DATA).

## Methods

### System Building

Here, we detail the operations required to prepare the binding partners, corresponding to steps 1 and 2 of Figure 1. Proteins must be preoriented before input; i.e., the center of the transmembrane domain of both binding partners is at the origin with the appropriate orientation given that the bilayer will be built parallel to the *x*–*y* plane. Such prealignment comes as standard for structures downloaded from the OPM server.^19^

(1) Structures are initially checked and, where necessary, repaired using the Modeller program.^20^ Specifically, the FASTA sequence of the protein is downloaded from the PDB database^2^ (placing the FASTA file in the folder is enough if there is no connection to the Internet), and this is used to patch up to 15 consecutive missing residues. Modeller will also place missing atoms. In its current form, the patching code can only handle two chains at most. This step can be skipped, but it is necessary for a complete simulation.
(2) The protein is immersed in a POPE bilayer and solvated via the PACKMOL-memgen tool^21^ available through the AmberTools(v.18+) package. Lipid and TIP3P water molecules are placed using a random seed, and 80 loops are performed during PACKMOL’s GENCAN routine to improve packing with a total of 120 nloops used for all-together packing. A tolerance of 2.4 Å is used to detect clashes between molecules. POPE residue names are then corrected to reflect the SLipid^24^ nomenclature before the topology files are generated through GROMACS.^22^ Since the SLipid and Amber14SB^23^ force fields use different angle and dihedral descriptions, a small fix is applied to allow the two to work in conjunction after this step. Finally, the system is neutralized by swapping water molecules for the appropriate number of Na^+^ or Cl^–^ counterions.

### Molecular Dynamics

Here, we provide details on the MD protocol used to simulate the binding partners, corresponding to step 3 of Figure 1. All simulations are run on the GROMACS^22^ MD engine, with Amber14SB^23^ and SLipid^24^ force fields used for the protein and lipids, respectively. The system is energy minimized using a steepest descent algorithm, with a tolerance threshold set to 200 kJ mol^–1^ nm^–1^. The initial step size is set to 1 pm and the maximum number of allowed steps to 5 × 10^6^. The cutoffs for both Coulombic and van der Waals interactions are set to 1.2 nm.

The protein is then equilibrated for 20 ns within an isothermal–isobaric ensemble; *T* is set to 310.15 K and pressure to 1 bar with a 2 fs step size. The constraint algorithm LINCS^26^ is applied to the bonds. A particle mesh Ewald summation is used to treat long-range interactions, and a velocity-rescale temperature with a coupling constant of 0.1 ps is applied separately to protein, lipids, and water/ions. A Berendsen pressure coupling method implemented semi-isotropically maintains the pressure with a coupling constant of 1.0 ps and compressibility of 4.5 × 10^–5^ bar^–1^. Velocities are randomly assigned from a Boltzmann distribution at *T*. A second equilibration stage is then run for 40 ns with the same settings but with all constraints removed. Finally, production occurs over a 10 ns time scale, for reasons shown in Figure S1, again in an isothermal–isobaric ensemble with the same settings as the equilibration. Atomic coordinates are saved every 5 ps and used to generate a STID map following the procedure outlined by Rudden and Degiacomi.^16^

### Homology Modeling

Several test cases only had their ligand and/or receptor starting structure known from a homologue, sometimes bound to an alternative binding partner. For these cases, receptor and ligand crystal structures were mutated into their target counterparts via the Modeler program.^20^ Motifs up to 15 residues long were permitted to be patched if they were missing from the structure, and structures were kept frozen to prevent optimization of models. The roto-translations returned by JabberDock were applied to these structures to yield the final predicted complexes. Table S1 reports on the sequence identity between homologues and the target structure. Their RMSD, determining case difficulty (see below), is also provided. We note that three benchmark cases (1ZOY, 2VT4, and 1EHK) feature binding partners extracted from a known complex that is a homologue to the target. Although not a real-world test case, these are suitable benchmark cases as the conformations of subunits in the two dimers differ.

### Protein Docking

Here, we provide details on the docking process of protein surfaces generated from STID maps, corresponding to steps 5, 6, and 7 of Figure 1. An initial starting point with the two input monomers’ transmembrane region centers of mass centered at the origin is used prior to generating any models. JabberDock uses a seven-dimensional space for implementation comfort when roto-translating the STID maps. Three dimensions define ligand translation in the Cartesian space. Three dimensions define an axis of rotation for this ligand, and one dimension defines a rotation angle around this axis. The *x* and *y* translation values are limited by the size of the receptor, and the ligand is only allowed to move ±5 Å along the *z*-axis. The axis of rotation is the *z*-axis, which is permitted to precess by up to 0.157 radians (9°) into the *x*–*y* plane. Possible rotation angles in radians range between 0 and 2π.

To navigate the potential energy surface (PES) associated with the scoring function and produce an ensemble of possible docked poses, JabberDock leverages a distributed heuristic global optimization algorithm featured in the POW^er^ optimization environment–particle swarm optimization “kick and reseed” (PSO-KaR).^25^ PSO-KaR is used to explore the PES over 300 iterations using 80 randomly initialized agents (“particles”). According to the “kick and reseed” procedure, particles converging to a local minimum (i.e., with a velocity decaying to less than 4% of the search space dimension in each direction) are randomly restarted, and a repulsion potential is placed at their convergence location. The whole optimization process is repeated three times, with the memory of previous repulsion potentials retained from one repetition to the next. In summary, this docking procedure requires the evaluation of 72,000 docking poses. To obtain a diverse ensemble of solutions, 300 poses were finally selected as representatives from the pool of poses having a positive score using a *K*-means clustering algorithm on the 7-dimensional coordinates associated with each model.

### Assessment of Models Accuracy

Following the CAPRI guidelines, we used three metrics to determine the quality of a model: the ratio of correct contact residues (a valid contact defined as an atom within 5 Å of the binding partner) to the number of residues in the predicted complex, *f*_nat_, the RMSD between the alpha carbons of the known crystal pose and the predicted pose, and the RMSD of the two poses between the α-carbons at the interface (defined as within 10 Å of the binding partner). CAPRI guidelines specify four levels of possible success criteria: (1) incorrect, where RMSD > 10.0 Å and interfacial RMSD > 4.0 Å OR *f*_nat_ < 0.1, (2) acceptable quality, where RMSD ≤ 10.0 Å or interfacial RMSD ≤ 4.0 Å and 0.1 ≤ *f*_nat_ < 0.3 OR *f*_nat_ ≥ 0.3 and RMSD > 5.0 Å and interfacial RMSD > 2.0 Å, (3) intermediate quality, where RMSD ≤ 5.0 Å or interfacial RMSD ≤ 2 Å and 0.3 ≤ *f*_nat_ < 0.5 OR *f*_nat_ ≥ 0.5 and RMSD > 1.0 Å and interfacial RMSD > 1.0 Å, and (4) high quality, where RMSD ≤ 1.0 Å or interfacial RMSD ≤ 1.0 Å and *f*_nat_ ≥ 0.5. The protocol for applying this list of inequalities follows the order provided, beginning with defining the incorrect predictions. In the text, we qualify the result of a test as of high, intermediate, or acceptable quality if at least one in the top 10 ranked models matches the criteria above.

### Case Difficulty Classification

Docking cases are classified under three levels of difficulty associated with their flexibility, which we quantify via the RMSD difference between the Cα atoms at the interface after superposing the bound and unbound interfaces. Cases can be classified as either rigid-body (or easy), medium, or difficult. Easy cases are those with a minimal difference between the unbound crystallized structures and the bound: less than 1 Å difference. In medium cases, the RMSD difference is between 1 and 2.5 Å. Finally, difficult cases can be anything greater than 2.5 Å. Thus, the difficult cases are accordingly significantly more challenging than the other two, particularly given that the requirements for an acceptable success are close to the upper boundaries that define the difficult cases. Our benchmark set featured two easy, 15 medium and three difficult cases, as detailed in Table S1, spreadsheet. The RMSDs reported in Table S1, spreadsheet refer to those between target structures and crystal structures, either of the unbound molecule or mutated structure from the homologue.

## Supporting Information Available

Information as mentioned in the text (PDF)
Information as mentioned in the text (XLSX)
