# Supplementary material for: Transmembrane Protein Docking with JabberDock
Source: J Chem Inf Model. 2021 Feb 26;61(3):1493–9. doi: 10.1021/acs.jcim.0c01315 (PMC8041277; doi:10.1021/acs.jcim.0c01315)
Supplement: Supplementary file 1 — ci0c01315_si_001.pdf [file ci0c01315_si_001.pdf]

Supporting Information

Transmembrane Protein Docking with JabberDock

Lucas S. P. Rudden [1], Matteo T. Degiacomi [1]*

[1] Department of Physics, Durham University, South Road, DH1 3LE, UK

* matteo.t.degiacomi@durham.ac.uk

Supplementary Data

Convergence of Side Chain Motion

The shape of a STID map depends on the length of the Molecular Dynamics (MD) simulation used to build it. In our previous work [1], we compared the Pearson cross-correlation coefficient (CCC) of STID maps generated from increasingly long trajectories and observed that this metric converged after 600 ps. This timescale represents the time required for the side chains of a protein to explore their local conformational space in a water solvent. Since side chain dynamics are slower in a membrane than in water, longer convergence times should be expected. Therefore, here we performed the same benchmark described above for the membrane proteins in our benchmark set to ensure that the surfaces used for docking are fully representative of their biphasic environment. As in our previous work, we assumed convergence using the same arbitrarily high CCC value of 0.9997, reported between only a STID map's non-zero values.

Figure S1(a) illustrates the evolution of CCC of consecutive STID (*i.e.* generated with increasingly long MD simulations) for 17 unique proteins used for docking. The CCC increases rapidly over a few ns, before asymptoting towards one. The Gaussian fitted in Figure S1(b) shows that, on average, CCCs converge in ~5.5 ns, but some require upwards of 8 ns. This simulation time is long enough to account for all side chain motions but short enough that no significantly large conformational changes can occur. Thus, we extract the last 9 ns of proteins simulated in a lipid bilayer to generate suitable STID maps.

Isosurface Cutoff Benchmark

In our previous work[1], we introduced the concept of applying a isovalue cutoff to STID map volumes to produce an isosurface representation suitable for docking. We first demonstrated that a value of 0.43 emerged naturally from a relationship between the Solvent Accessible Surface Area (SASA) of a protein and the average STID value. Then, we also independently demonstrated that this cutoff

value provided the highest score at the known binding site *versus* other cutoffs. Since this value was derived for proteins in solution, we performed a similar benchmark here. In Figure S2 we show the variation in the STID score with cutoff in the known binding site, normalised and averaged across all 20 cases in our benchmark set. Again, we find a value of 0.43 to be most appropriate to return the highest quality results.

Scoring Function Smoothness

To demonstrate the utility of our surface-based scoring function over more atomistic energy profiles, for all cases in our benchmark set we generated 100 new poses by applying small random perturbations (<2.5 Å) of ligands position from their known bound state. We then evaluated their STID map-based score, as well as their atomistic and coarse-grained van der Waals energies. The coarse-grained vdW score was calculated from the backbone atoms, with typical Martini parameters[2] used for the forcefield. We finally compared all these scores with their value at the known bound state, reporting them in terms of a percentage increase or decrease. The results of this comparison are shown for three examples in Figure S3. The STID score provided distinguishable gradients to the binding site, with a minimum at the ground truth. In contrast, the atomistic vdW energy has no discernable trends, with positions away from the binding site having higher or lower in energy. Furthermore, these energies featured extremely steep gradients, requiring the use of a log-scale for a manageable comparison. The coarse-grained vdW score features a smooth gradient to the binding site for the 3KCU case, but returns a similar result to the atomistic vdW score, albeit significantly less steep, for the other two. van der Waals scores for all proteins in our dataset behave consistently with the examples shown here. Our STID score behaves as exemplified for 15 out of 20 cases. In the remaining five cases a gradient is not clearly distinguishable, although percentage increases always remain in the same order of magnitude as demonstrated here.

Quantifying the 'flatness' of a binding interface

We find that most unsuccessful cases feature binding interfaces devoid of ‘feature-rich’ regions on the STID isosurfaces. By this, we mean that the surface is relatively flat, with an absence of ‘bumpiness’. The ability of JabberDock to consider flat surfaces is further hindered when a docked pose features some minor shape intersection, which nullifies subtle detail on the surface. We are currently working on improving our scoring function to better consider the topography of the isosurface at this scale. We illustrate two different surfaces; one with a flat surface, that of wild type cytochrome c oxidase (PDB: 1M56), and one with a bumpier surface, that of cytochrome b6f (PDB:1Q90) in Figure S4. This bumpiness is caused by both the flexibility of the backbone, and the diversity of amino acids present at the binding site – with a variation of those with short and long side-chain resulting in more topographically distinct surfaces. Since the level of bumpiness can be considered subjective, we quantified it by measuring local regions' similarity on the surface to a plane.

To quantify this, we first isolated the binding site STID map from the rest of the protein (indicated in red in Figure S4), which we define as residues within 5 Å of the binding partner, for all proteins in our dataset. We then tessellated the surface at our optimised cutoff of 0.43 with a series of triangles using a marching cubes Lewiner algorithm. Each triangle has a surface area of 0.5 $Å^2$. For every triangle’s normal to the surface, we calculate the mean dot product with all other neighbouring triangle normals over a 10 $Å^2$ surface area as a measure of the local bumpiness value (B). The mean of all localised B gives the overall bumpiness of the binding site surface. A value closer to 1 indicates that the surface is more planar, while 0 is more spherical. Table S2 provides the corresponding B for each test case. We find that the majority of proteins involved in cases that succeeded (*i.e.* a successful model in the top 10) return a lower B value overall (B = 0.74(2), averaged across all successful models), while cases which were not so successful feature more planar surfaces (B = 0.78(1)). Exceptions to this rule include that of 3RW0, which was not successful due to the binding site being

occluded in the STID isosurface of the receptor. Thus, while the bumpiness of a STID isosurface is important, it is not the sole cause of unsuccessful cases, and *vice versa*.

Computational Load

Molecular Dynamics (MD) simulations were run with GROMACS 2016, on a single GPU, using 2 CPUs and 4 OpenMP threads. The specific hardware used included an NVIDIA Tesla K40m GPU, an Intel® Xeon® CPU E5-2650 v2 @ 2.60Ghz, and 64 GB of DDR4 RAM clocked at 2133 MHz. The MD simulation time required to equilibrate the system is longer when a membrane is involved. On average, a complete MD cycle (building the system, energy minimisation, equilibration, production, and parsing the simulation data) took approximately three days. Depending on the size of the system, the conversion of the last 9 ns of MD trajectory into a STID map then took roughly 30 minutes and 20 GB of RAM.

Each POW^{er} docking case was run using MPI on 12 Intel® Xeon® CPU E7-4809 v2 @ 2.1 GHz, across 600 GB of memory and a 1 × Intel TrueScale 4× QDR single-port InfiniBand interconnect. Docking runtimes were faster for membrane proteins than for soluble ones, owing to smaller protein sizes and reduced search space. Each docking run took approximately 12 hours and required 10 GB of RAM.

Table S2: Bumpiness of each protein (both receptor and ligand if different), with the chain indicated in the parentheses and the rank of the first successful model (if applicable). The average for successful test cases is $B = 0.74(2)$, while that of unsuccessful models is $B = 0.78(1)$. Method described in SI.

PDB	Bumpiness, *B*	First successful model
1K4D (C)	0.777(2)	2
3S33 (A)	0.752(1)	1
3S33 (B)	0.741(1)	1
1GU8 (A)	0.737(2)	1
2F95 (B)	0.751(2)	1
3V3C (A)	0.807(1)	1
3A7K (A)	0.742(2)	2
3OMI (A)	0.769(1)	X
1QLE (C)	0.771(1)	X
2ZT9 (A)	0.724(2)	5
1YQ3 (C)	0.765(2)	159
1YQ3 (D)	0.770(3)	159
1ZRT (C)	0.743(2)	3
1YEW (B)	0.739(2)	8
3KCU (A)	0.746(2)	5
3ODU (A)	0.805(2)	X
3RW0 (A)	0.759(2)	X
4EA3 (A)	0.755(1)	1
2IC8 (A)	0.736(2)	1
2Y00 (A)	0.759(2)	8
2Y00 (B)	0.756(1)	8
3Q7K (A)	0.775(1)	1
1C8S (A)	0.715(2)	7
2RMZ (A)	0.714(1)	1
2K1A (A)	0.722(3)	1
2N2A (A)	0.768(4)	135
2M0B (A)	0.785(2)	135

Supplementary Figures

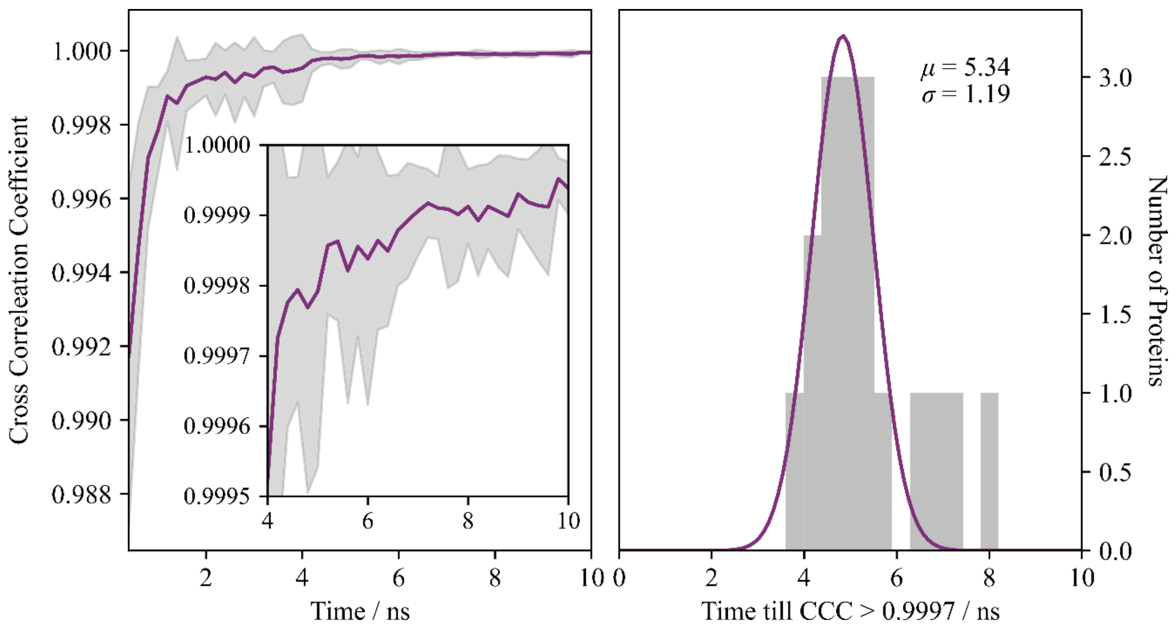

Figure S1: (left) Cross-Correlation Coefficient (CCC) between successive STID maps built from increasingly long timescales. The palatinate line indicates the average of 17 unbound membrane proteins used for docking, the grey region represents the standard deviation at each trajectory length. The inset is a zoom into the top 4% of the figure. The CCC is calculated between non-zero values. (b) The number of proteins achieving a consistent CCC of 0.9997 by the listed time. A Gaussian (palatinate) has been fitted to the histogram. All protein motion converged within 9 ns, with the majority in the first 5 ns. The mean (μ) and standard deviation (σ) are reported.

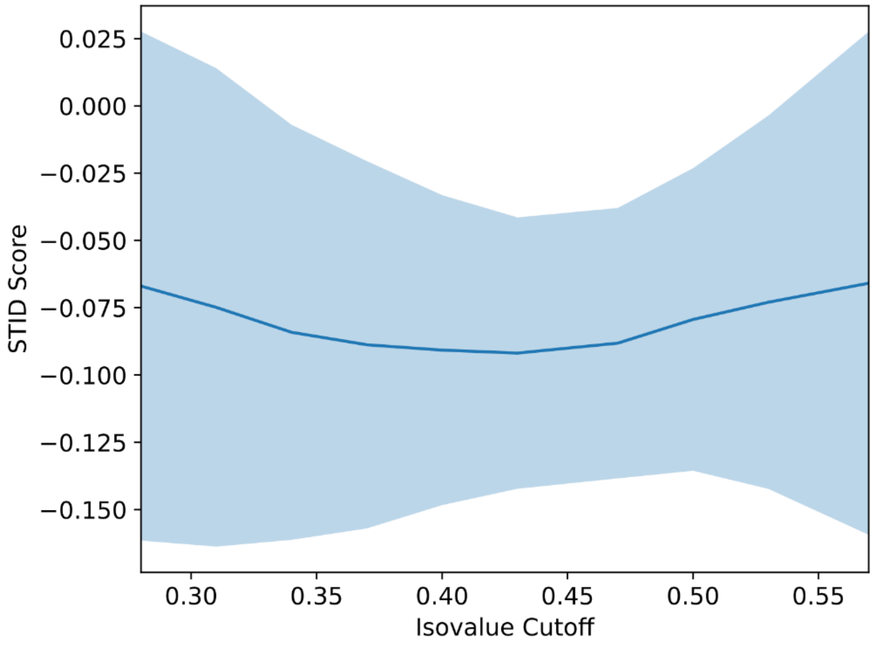

Figure S2: Mean variation in normalised STID score across the 20 protein cases with isovalue cutoff choice. The dark central line indicates the mean, the shaded region the standard deviation. The minimum (indicating what on average yields the best shape complementarity) occurs at a cutoff of 0.43.

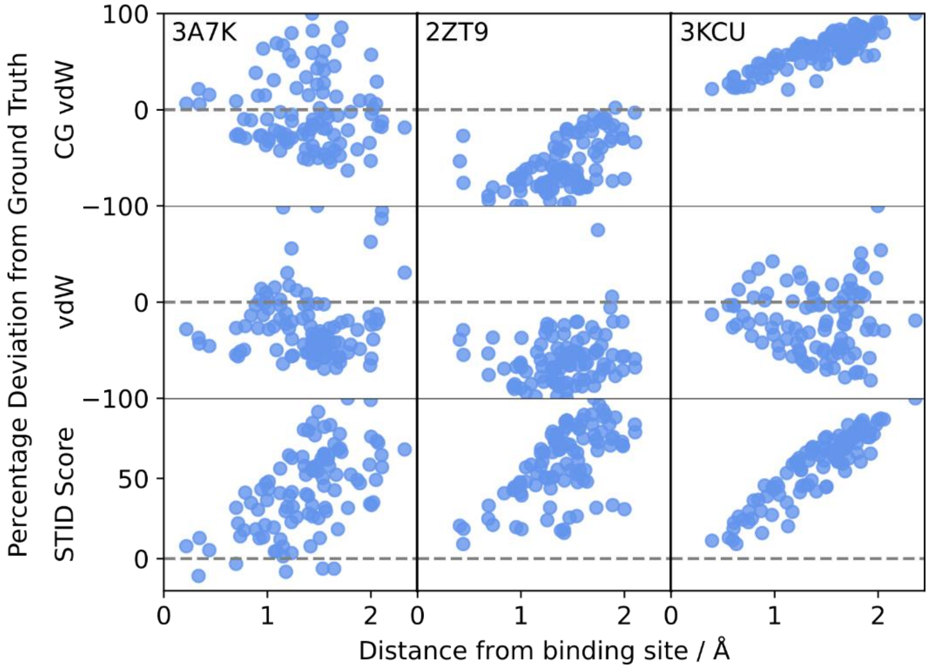

Figure S3: Comparison between atomistic docking scores, represented by coarse-grain (top) and all-atom (middle) van der Waals energy, and our STID map-based score (bottom). For three different transmembrane protein complexes, we perturb the position of the known docked pose, reporting the percentage deviation of scores from their value at the bound state. For clarity, percentage for atomistic van der Waals energies are plotted in a log-scale. Our STID score produces clearer and distinctly less sharp gradients.

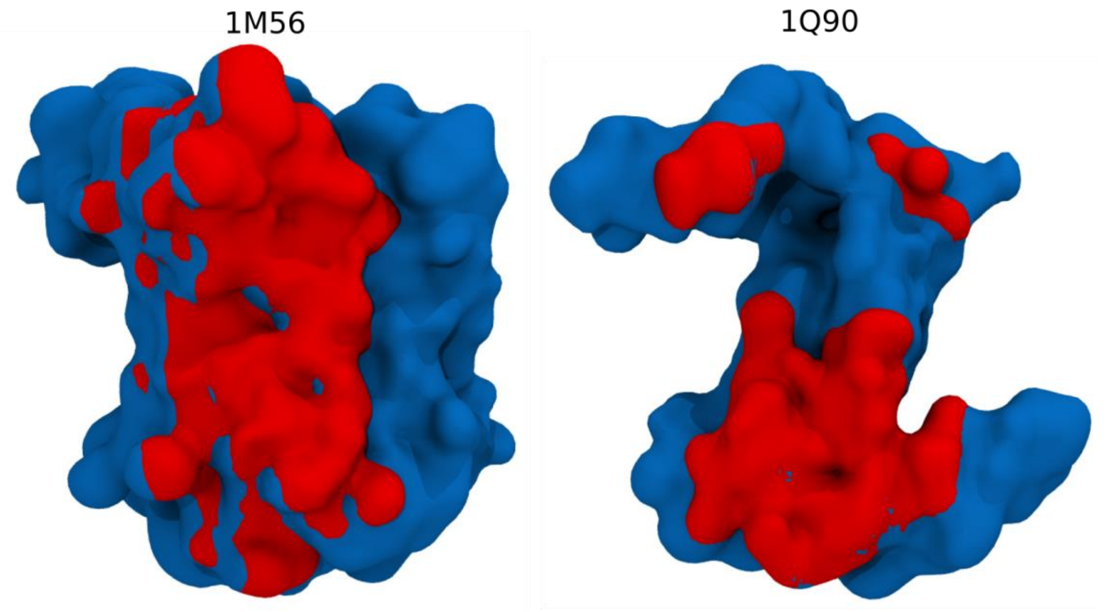

Figure S4: STID map isosurface at 0.43 cutoff for two cases, with their binding site indicated in red. (left) Wild type cytochrome c oxidase (PDB: 1M56) receptor, with B = 0.769(1).JabberDock was unable to identify a single acceptable model for this case. (right) Cytochrome b6f (PDB:1Q90) receptor, with B = 0.724(2). JabberDock identified an intermediate quality result at rank 5 for this complex.

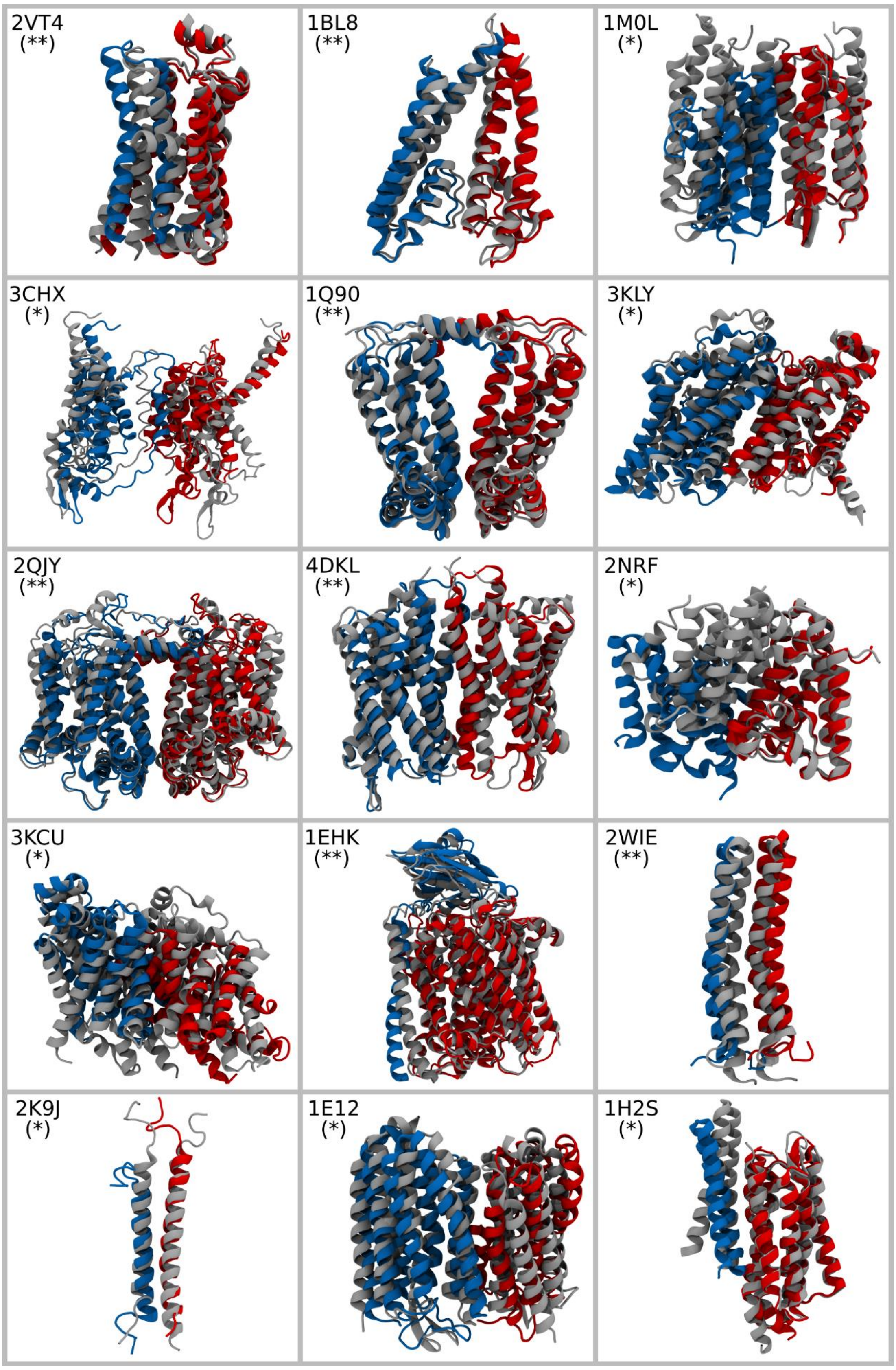

Figure S5: Best model in the top 10 for every successful case (noted as * if acceptable, and ** if intermediate). The known bound state (indicated by its PDB code) is shown in grey, the receptor and ligand used for docking in red and blue respectively. See Table S1 for details.

Supplemental Bibliography

(1) Rudden, L. S. P.; Degiacomi, M. T. Protein Docking Using a Single Representation for Protein Surface, Electrostatics, and Local Dynamics. *J. Chem. Theory Comput.* **2019**, *15* (9), 5135–5143. https://doi.org/10.1021/acs.jctc.9b00474.

(2) De Jong, D. H.; Singh, G.; Bennett, W. F. D.; Arnarez, C.; Wassenaar, T. A.; Schäfer, L. V.; Periole, X.; Tieleman, D. P.; Marrink, S. J. Improved Parameters for the Martini Coarse-Grained Protein Force Field. *J. Chem. Theory Comput.* **2013**, *9* (1), 687–697. https://doi.org/10.1021/ct300646g.
